# Leveraging Digital Methods to Engage Sexual and Gender Minority Caregivers of People With Dementia

**DOI:** 10.1093/geroni/igaa057.2775

**Published:** 2020-12-16

**Authors:** Joel Anderson, Jason Flatt, Jennifer Jabson Tree, Alden Gross, Karen Rose

**Affiliations:** 1 University of Tennessee-Knoxville, Knoxville, Tennessee, United States; 2 University of Nevada, Las Vegas, School of Public Health, Las Vegas, California, United States; 3 University of Tennessee, Knoxville, Tennessee, United States; 4 Johns Hopkins Bloomberg School of Public Health, Baltimore, Maryland, United States; 5 Ohio State University, Colombus, Ohio, United States

## Abstract

Digital methods are a way to engage marginalized populations, such as sexual and gender minority (SGM) adults. No study to date has leveraged these methods to engage SGM caregivers of people with dementia. We used digital methods to access SGM caregivers of people with dementia in our study of psychosocial measures of caregiving for recruitment and data collection. Posts on social media and online registries targeted SGM caregivers. The study landing page received 2201 views; 285 caregivers completed the survey. Participants learned of the study most frequently from Facebook (45%). The sample was 84% white, with gay (52%), lesbian (32%), bisexual (11%), and other sexual orientations (5%) and transgender (17%) caregivers represented. While we exceeded goals for inclusion of Latinx (26%) and Native American (4%) caregivers, the number of African American SGM caregivers was lower than projected (7%). Digital methods are effective for engaging SGM caregivers of people with dementia.

